# Prediction of instantaneous perceived effort during outdoor running using accelerometry and machine learning

**DOI:** 10.1007/s00421-023-05322-0

**Published:** 2023-09-29

**Authors:** Cristina-Ioana Pirscoveanu, Anderson Souza Oliveira

**Affiliations:** 1 Department of Health Science and Technology, Aalborg University, Gistrup, Denmark; 2 Department of Materials and Production, Aalborg University, Fibigerstræde 16, Building 4, 9220 Aalborg Øst, Denmark

**Keywords:** Running, Fatigue, RPE, Borg, Wearable sensors, Machine learning

## Abstract

The rate of perceived effort (RPE) is a subjective scale widely used for defining training loads. However, the subjective nature of the metric might lead to an inaccurate representation of the imposed metabolic/mechanical exercise demands. Therefore, this study aimed to predict the rate of perceived exertions during running using biomechanical parameters extracted from a commercially available running smartwatch. Forty-three recreational runners performed a simulated 5-km race on a track, providing their RPE from a Borg scale (6–20) every 400 m. Running distance, heart rate, foot contact time, cadence, stride length, and vertical oscillation were extracted from a running smartwatch (Garmin 735XT). Machine learning regression models were trained to predict the RPE at every 5 s of the 5-km race using subject-independent (leave-one-out), as well as a subject-dependent regression method. The subject-dependent method was tested using 5%, 10%, or 20% of the runner’s data in the training set while using the remaining data for testing. The average root-mean-square error (RMSE) in predicting the RPE using the subject-independent method was 1.8 ± 0.8 RPE points (range 0.6–4.1; relative RMSE ~ 12 ± 6%) across the entire 5-km race. However, the error from subject-dependent models was reduced to 1.00 ± 0.31, 0.66 ± 0.20 and 0.45 ± 0.13 RPE points when using 5%, 10%, and 20% of data for training, respectively (average relative RMSE < 7%). All types of predictions underestimated the maximal RPE in ~ 1 RPE point. These results suggest that the data accessible from commercial smartwatches can be used to predict perceived exertion, opening new venues to improve training workload monitoring.

**Supplementary Information:**

The online version contains supplementary material available at 10.1007/s00421-023-05322-0.

## Introduction

Endurance exercises such as cycling and running are widely practiced for leisure, health management, and improving athletic performance (Grunseit et al. 2018). Running especially has grown in popularity and more recreational practitioners are increasingly participating in races ranging from 5 to 21 km. Regardless of the fitness level, runners are subjected to the effects of fatigue during their training sessions and races, reducing their performances due to metabolic and neuromuscular factors (Martin et al. 2010; Oliveira et al. 2013). Fatigue influences running mechanics, such as increasing the peak ground reaction forces and loading rates (Christina et al. 2001; Jafarnezhadgero et al. 2019; Luo et al. 2019; Pirscoveanu et al. 2021), which may be linked to the increased accumulation of metabolites (blood lactate, inorganic phosphates, K +) (Boyas and Guével 2011; Gholami et al. 2020). Moreover, heart rate is a relevant indicator of fatigue, as fatigue overloads the cardiovascular system due to the reduced efficiency in generating energy and metabolite removal (Schneider et al. 2018).

Fatigue progression has also been assessed through ratings of perceived exertion (Jo and Bilodeau 2021), defined as a psycho-physiological marker of intensity that combines subjective feelings of effort, strain, discomfort, and/or fatigue experienced during exercise (Marotta et al. 2021; Cheval and Boisgontier 2021). Amongst the different types of RPEs, the 6–20 Borg scale, which ranges from 6 (no exertion) to 20 (maximal exertion), is widely used in running exercises to indirectly measure fatigue levels (Borg 1970, 1990). Previous studies using the 6–20 Borg scale set a “13” RPE as a moderate intensity and a “17” RPE as exercise failure to terminate fatiguing protocols (Koblbauer et al. 2014; Jafarnezhadgero et al. 2019; Borgia et al. 2022). Moreover, RPE has shown promising results to define running training loads (Chai et al. 2019). Therefore, implementing RPE as an index of fatigue and/or marker for training loads is highly relevant.

RPE is becoming more relevant within the running community, but RPE is a subjective measure influenced by external factors other than the running exercise itself. Effort perceptions vary widely across individuals, and the real instantaneous exertion can be overestimated for women and underestimated for regular running practitioners (Skatrud-Mickelson et al. 2011). Moreover, training status might affect RPE in older adults and lead to overestimated exertion in untrained individuals (Jabbour and Majed 2018). Therefore, the use of RPEs to determine fatigue levels might be limited due to the subjective nature of the metric.

Since RPE is based on a physical exercise that generates measurable variables, it is plausible that a set of data from the exercise can provide objective information to predict the RPE itself. Machine learning is being increasingly used in sports, as sports performance is relying more on objective data within the last decades. There are several studies applying machine learning in running to predict impact loading/ground reaction forces (Girka et al. 2020; Oliveira et al. 2022), running velocity (Wiecha et al. 2022), and terrain types a runner is exposed to (Dixon et al. 2019). Moreover, fatigue during running has been investigated using metabolic (Bustos et al. 2022) and mechanical variables (Gholami et al. 2020; Marotta et al. 2021). However, assessing relevant data for fatigue prediction during outdoor activities might demand users to use additional wearables, limiting the audience's interest in the technology. Interestingly, current wearable technologies used for fitness tracking offer measurements of running biomechanical parameters such as running speed, stride length, foot contact time, and vertical oscillation with reasonable validity (Price et al. 2017; Støve et al. 2019; Evenson and Spade 2020; Carrier et al. 2020). Therefore, it may be possible to use post-processed running biomechanical parameters from commercial smartwatches to predict the fatigue state of a runner while performing the activity.

In this study, the aim is to create a regression model to accurately predict instantaneous 6–20 Borg scale using biomechanical data from runners performing a simulated 5-km race. The biomechanical input data for the prediction is attained from a commercial smartwatch. We hypothesized that it is possible to create regression models to predict RPE within ± 1 Borg point from a limited subset of features extracted from the smartwatch. The confirmation of our hypothesis is a strong contribution to the field of exercise sciences, as such types of prediction models can be incorporated into wearables to alert runners about their physical exertion while exercising.

## Methods

Forty-three recreational runners (30 males, 13 females, age: 25 ± 3 years, height: 180 ± 16 cm, weight 82 ± 20 kg) volunteered to participate in the study. The sample size in this study is in accordance with current recommendations for the conduction of running biomechanics studies (Oliveira and Pirscoveanu 2021) and may provide sufficient data for machine learning regression model predictions. The group presented 8 ± 4 years of running experience and a weekly running volume of 24 ± 15 km. Their self-reported 5-km race pace was 4:40 ± 0:38 min/km. Inclusion criteria included the current practice of running training protocol and being injury-free for a minimum of 6 months before the test. Participants were asked to avoid performing any strenuous exercise 24 h before the test, as well as avoid consuming caffeine and alcohol within the 12 h preceding the experiment. Participants were verbally informed about the experimental procedure and provided verbal and written informed consent to participate in this study. The local ethical committee (Region Nordjylland, Denmark) approved the procedures applied in the study, and all methods were carried out in accordance with relevant guidelines and regulations from the Declaration of Helsinki (2004).

### Experimental design

**Fig. 1:**
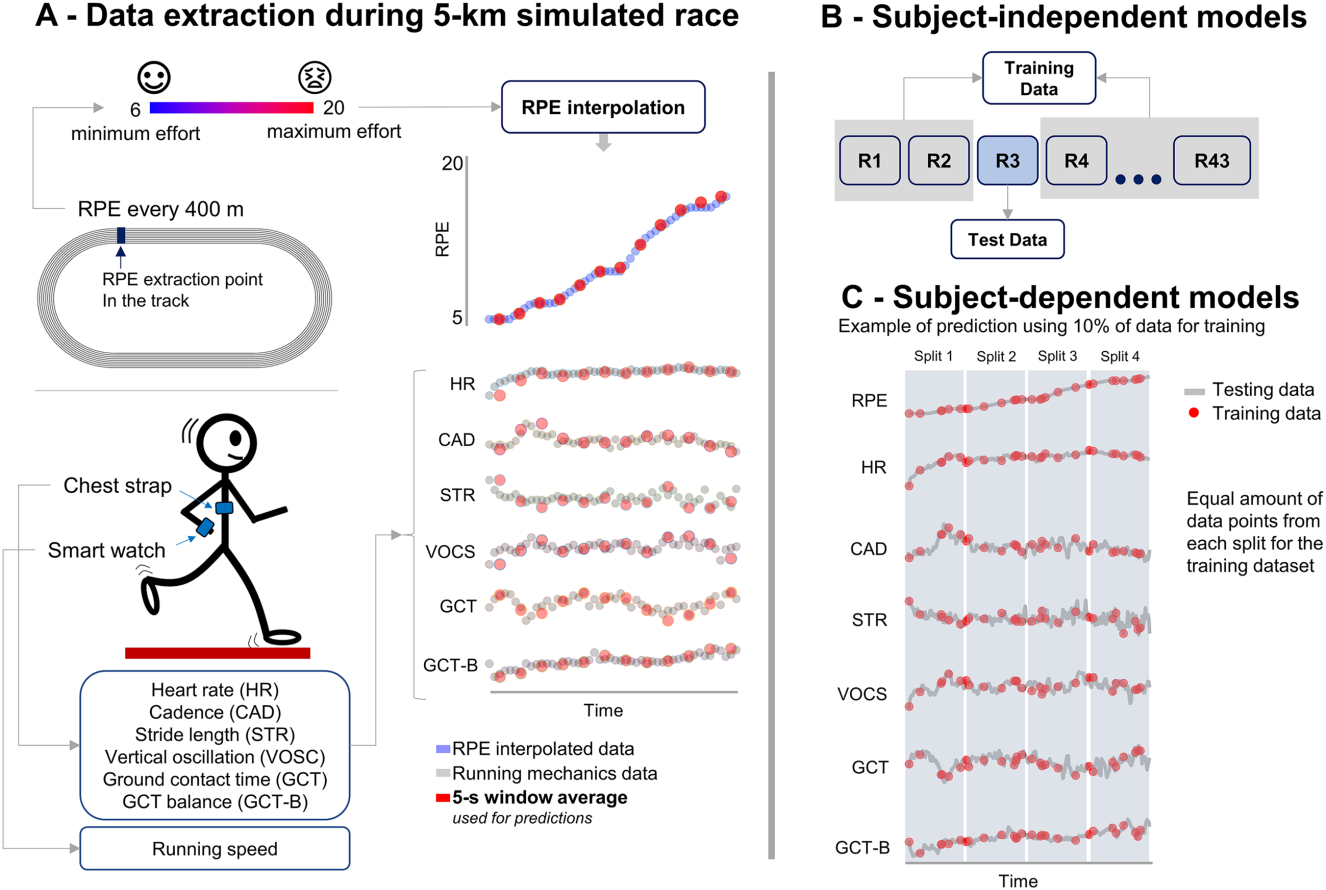
Data acquisition and analysis. In **A**, the rating of perceived exertion (RPE) data from 5 km simulated running was extracted at every 400 m, being subsequently interpolated to match the same number of samples in the running biomechanical variables. Both RPE and running mechanics data were reduced to 5-s windows for machine learning predictions. In **B**, the subject-independent machine learning models were applied using a leave-one-out approach where all runners (marked as “R”) but one were used for training the model. The excluded runner was the test dataset. In **C**, the subject-dependent machine learning models were applied by splitting the dataset of a given subject into a training set and a test set. The test set comprised 5%, 10%, or 20% of the total amount of data of the runner (the figure exemplifies 10% of the total data). A similar amount of test data was extracted from four different splits of the dataset (0–25%, 26–50%, 51–75%, and 76–100%), assuring balanced exposure to biomechanical behavior across the entire exercise

In a single session, participants were initially provided with a 10-min warm-up consisting of 2 laps on a 400-m outdoor running track, walking lunges, running with high knees, and leg swings (Pirscoveanu et al. 2021). Subsequently, runners were asked to perform a simulated running race until exhaustion on the running track using their preferred regular running shoes. Runners were asked to maintain a stable running speed based on the reported 5-km race pace throughout the test. The running speed was measured continuously through the embedded GPS on the smartwatch. Moreover, the consistency of the running speed throughout the test was assured by the experimenters checking if the runner was within ± 3 s of the expected time at every 200. RPE was assessed at the end of every 400 m using the 6–20 Borg scale. The test was terminated when the runner could not maintain a constant speed in two consecutive speed checks. Therefore, some participants exceeded the 5 km distance requirement. Figure 1A illustrates the experimental design and the variables acquired during the experiments.

### Data acquisition and analysis

A commercial smartwatch (Garmin Forerunner 735XT, Garmin International, Kansas City, MO) was used to assess running speed, heart rate, and running biomechanical parameters as described elsewhere (Pirscoveanu et al. 2023). A compatible chest strap containing heart rate sensors and a tri-axial accelerometer was used to acquire instantaneous heart rate and trunk accelerometry. The data from both heart rate sensors and accelerometry cannot be accessed for customized data processing. However, the smartwatch utilizes data processing algorithms that outputs heart rate and running biomechanical parameters such as running cadence, running speed, vertical oscillation, stride length, foot contact time, and foot contact time symmetry. All smartwatch data were sampled at 1 Hz. The validity of this type of device has been previously verified for the assessment of heart rate (Price et al. 2017; Støve et al. 2019), as well as running cadence, ground contact time, and vertical oscillation (Adams et al. 2016; Carrier et al. 2020). The data processing was performed using custom-made scripts (Matlab 2020b, The Mathworks Inc., Natick, MA, USA). The RPE values were interpolated between laps with a 1 Hz frequency to match the number of samples extracted from the running parameters. The heart rate was normalized by the maximum expected heart rate using previous literature (Tanaka et al. 2001). Subsequently, the biomechanical and RPE data from all runners were reduced to averaged points for every 5 s.

### Machine learning—model validation

The regression learning app from Matlab (Matlab 2020b, The Mathworks Inc., Natick, MA, USA) was used to run several machine learning algorithms using a fivefold validation method. Feature scaling was applied to each feature to prevent the feature magnitude from affecting the learning process. Scaling of features to:

$$\left[0, 1\right]: {x}^{\prime}=\frac{x - \mathrm{mean}\left(x\right)}{\mathrm{max}\left(x\right)-\mathrm{min}\left(x\right)},$$

where *x* is the original value and *xʹ* is the scaled value.

We allocated the data from 26 runners (~ 60% of the sample) to validate machine learning models in the three different datasets. We first trained and validated models to predict the RPE using the runner’s age, body mass, and body height, as well as the running biomechanical data (running distance, heart rate, cadence, vertical oscillation, stride frequency, ground contact time, and ground contact time symmetry). Running speed was excluded from the training models since it was set to be a constant value across the experiment. The training models using all ten variables presented the best performance (Supplementary Fig. 1, white bars). However, the implementation of a leave-one-out design to predict the RPE from a single individual cannot include invariant features such as age, body mass, and body height, since they have no variability across the running trial. Therefore, two other models were validated: a model with only biomechanical variables except for running speed (Supplementary Fig. 1, gray bars) and a model with only biomechanical variables except running speed and running distance (Supplementary Fig. 1, black bars). This third model was tested since running distance is a cumulative variable that may highly correlate with RPE.

We found that the inclusion of all variables resulted in the lowest prediction errors, followed by the models using biomechanical data except running speed. As expected, removing the running distance from the training dataset substantially increased the prediction errors regardless of the applied algorithm. Moreover, classic linear models such as linear regression and linear support vector machine presented substantial errors > 1 RPE, whereas the best-performing model that included running distance (gray bars) was the Gaussian process regression (GPR, rational quadratic) with an error = 0.22. Therefore, further RPE predictions were conducted using the GPR rational quadratic algorithm, including all biomechanical variables except running speed. The GPR applies Bayesian non-parametric regressions to compute joint multivariate Gaussian posterior distributions of a test set when given a training set (Schulz et al. 2018). The GPR can be advantageous for its ability to make predictions using fewer parameters.

### Machine learning—subject-independent model

Predictions of RPE over time were processed using a leave-one-out approach (Fig. 1B), where the training dataset consisted of all available data, except for the participant being tested (e.g., 42 participants in the training set, 1 participant in the testing set). The leave-one-out approach assures that no RPE information from the predicted participant was included in the training data (Derie et al. 2020).

### Machine learning—subject-dependent model

Subject-dependent models trained a personalized model for each runner, albeit only using data from that runner. Such type of model may be relevant for individualized predictions in case generalized subject-independent models do not provide sufficient accuracy. The data from a given runner was split into four-time sectors (0–25%, 26–50%, 51–75%, and 76–100% of the total amount of data). The data split into four sectors assured a balanced amount of data points throughout the − 5-km simulated race (Fig. 1C). Three subject-dependent models were evaluated in this study, allocating random samples of 5%, 10% or 20% of each data split into the model test set. Models that require only a fraction of the data but still provide acceptable accuracy may be more relevant to implementing the technology in real-time settings. The prediction of RPE was repeated ten times for each runner in each of the three test set dimensions to increase the variability of the test datasets. The final prediction accuracy result was an average across the ten predictions for each runner.

### Model evaluation and statistical analysis

The prediction quality from the proposed models to predict RPE using running biomechanical data was tested using Pearson’s correlation coefficient (*r*), absolute root-mean-square error (RMSE), and relative RMSE (rRMSE). The real and predicted RPE from the subject-independent models were averaged from five sequential measurements (~ 20 s of continuous recordings) at 25%, 50% 75%, and 100% of the total running time from each runner. Subsequently, the real and predicted RPEs in each running time percentage were compared using Bland–Altman plots displaying mean biases and the limits of agreement (e.g., 95% confidence interval). Moreover, the two-tailed *t* Student test and the respective Cohen’s *D* effect size (“small” values around 0.2, “medium” for 0.5, and “large” above 0.8 (Durlak 2009) were computed for each pairwise comparison. Regarding the subject-dependent models, we assessed the effect of the RPE extraction method (real vs predicted with 5%, 10%, and 20% training data) on the RPE at 25%, 50%, 75%, and 100% of the total running time using a one-way ANOVA, with Bonferroni pairwise post hoc tests if necessary. Bland–Altman plots were also generated for the pairwise comparisons of the subject-dependent models (shown in Supplementary Fig. 2 and Supplementary Table 1) The significance level for all statistical tests was set at *p* < 0.05.

## Results

The average time to complete the 5-km simulated race was 24.5 ± 6.8 min, in which the total running distance was 5.3 ± 1.3 km. The average heart rate was 93 ± 4.9% max HR, while the peak heart rate was 97.7 ± 4.7% max HR. Regarding RPE, the average perceived exertion was 15.2 ± 1.2, whereas the peak RPE was 19.6 ± 0.58. Moreover, a final dataset consisting of 12,514 samples from the 43 runners was used for machine learning predictions.

### Subject independent model

**Fig. 2:**
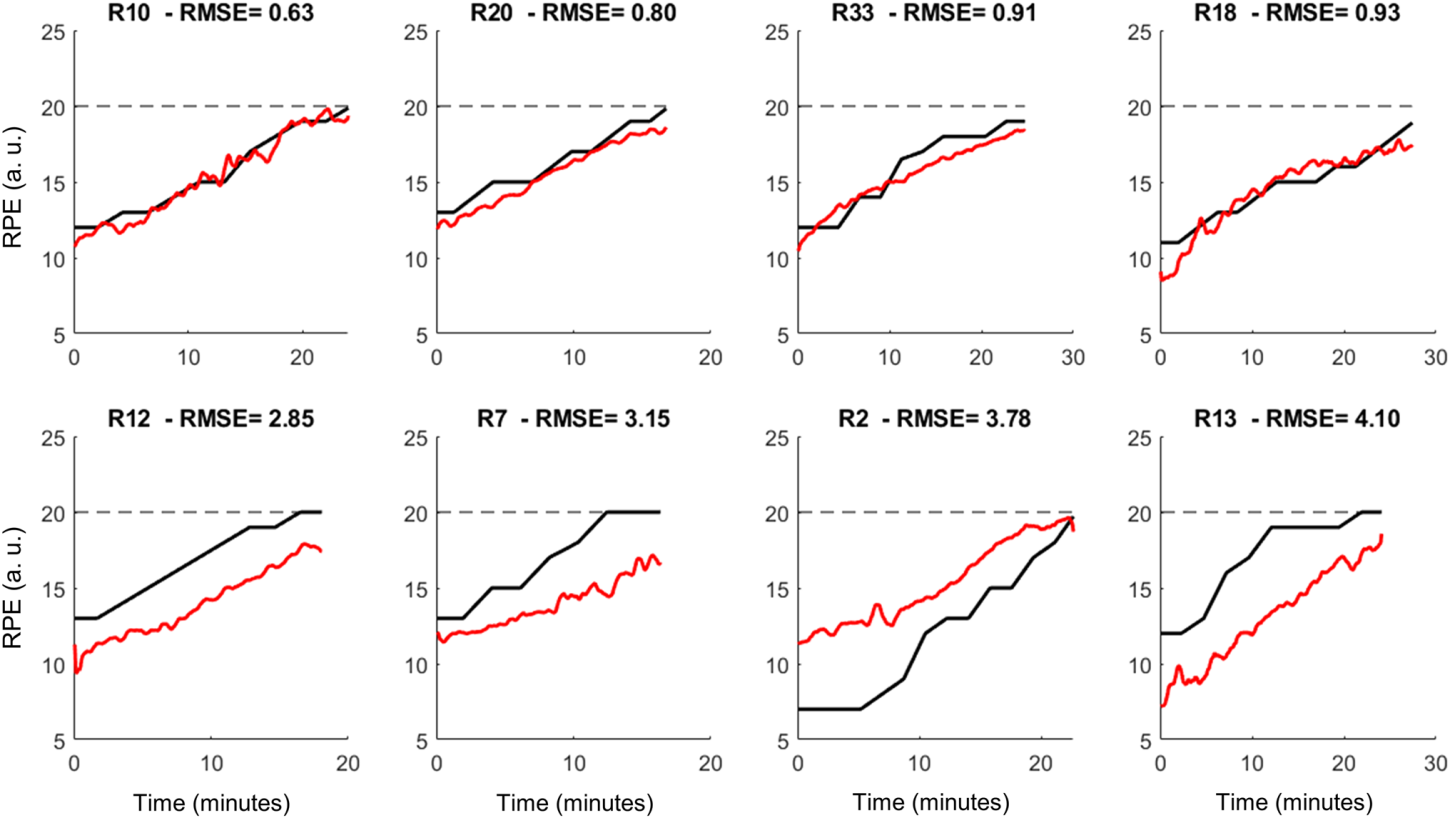
Real (black lines) and predicted rating of perceived exertion (RPE, red lines) during a 5-km simulated race from the four best predictions (top row) and the four worst predictions (bottom row)

Figure 2 illustrates some examples of RPE predictions. Predictions with low error throughout the exercise (< 1 a.u.) are presented in the top row, demonstrating high-quality prediction. For high-quality predictions, both real and predicted RPE present similar growing patterns over time. On the other hand, low-quality predictions may reach high errors (> 3 a.u.), for which a lack of similarity in the RPE patterns is noticeable due to extended periods of identical RPE values during real measurements. In addition, a general offset between real and predicted RPEs is noticeable in some runners, being predominantly an underestimation of the RPE (see R12, R7, and R13 in Fig. 2). The steady RPE values were presented either at the start or the end of the 5-km race.

In general, the association between real and predicted RPE was excellent (mean *r* > 0.9, Table 1), with an average RMSE < 2 RPE points, but with high inter-subject variability as the RMSE ranges from 0.63 to > 4 RPE points. The relative RMSE was on average < 12%, but it also reached > 30% for one runner.

**Table 1:** Pearson’s correlation coefficient, root-mean-square error (RMSE) and relative RMSE (rRMSE) from the prediction of ratings of perceived exertion (RPE) using biomechanical parameters during running using subject-independent and subject-dependent models

	Median	Average ± SD	Min	Max
*Subject independent model (leave-one-out)*				
Pearson’s correlation (*r*)	0.92	0.91 ± 0.05	0.71	0.97
RMSE (a.u.)	1.54	1.80 ± 0.81	0.63	4.10
rRMSE (%)	9.8	11.97 ± 5.89	4.11	31.42
*Subject-dependent model (5% training data)*				
Pearson’s correlation (*r*)	0.91	0.90 ± 0.04	0.74	0.95
RMSE (a.u.)	0.90	1.00 ± 0.31	0.53	1.74
rRMSE (%)	5.64	6.62 ± 2.39	3.28	12.53
*Subject-dependent model (10% training data)*				
Pearson’s correlation (*r*)	0.95	0.95 ± 0.01	0.89	0.94
RMSE (a.u.)	0.58	0.66 ± 0.20	0.41	1.20
rRMSE (%)	3.87	4.38 ± 1.56	2.59	8.43
*Subject-dependent model (20% training data)*				
Pearson’s correlation (*r*)	0.97	0.97 ± 0.01	0.94	0.99
RMSE (a.u.)	0.39	0.45 ± 0.13	0.29	0.76
rRMSE (%)	2.62	2.98 ± 1.03	1.80	5.86

**Fig. 3:**
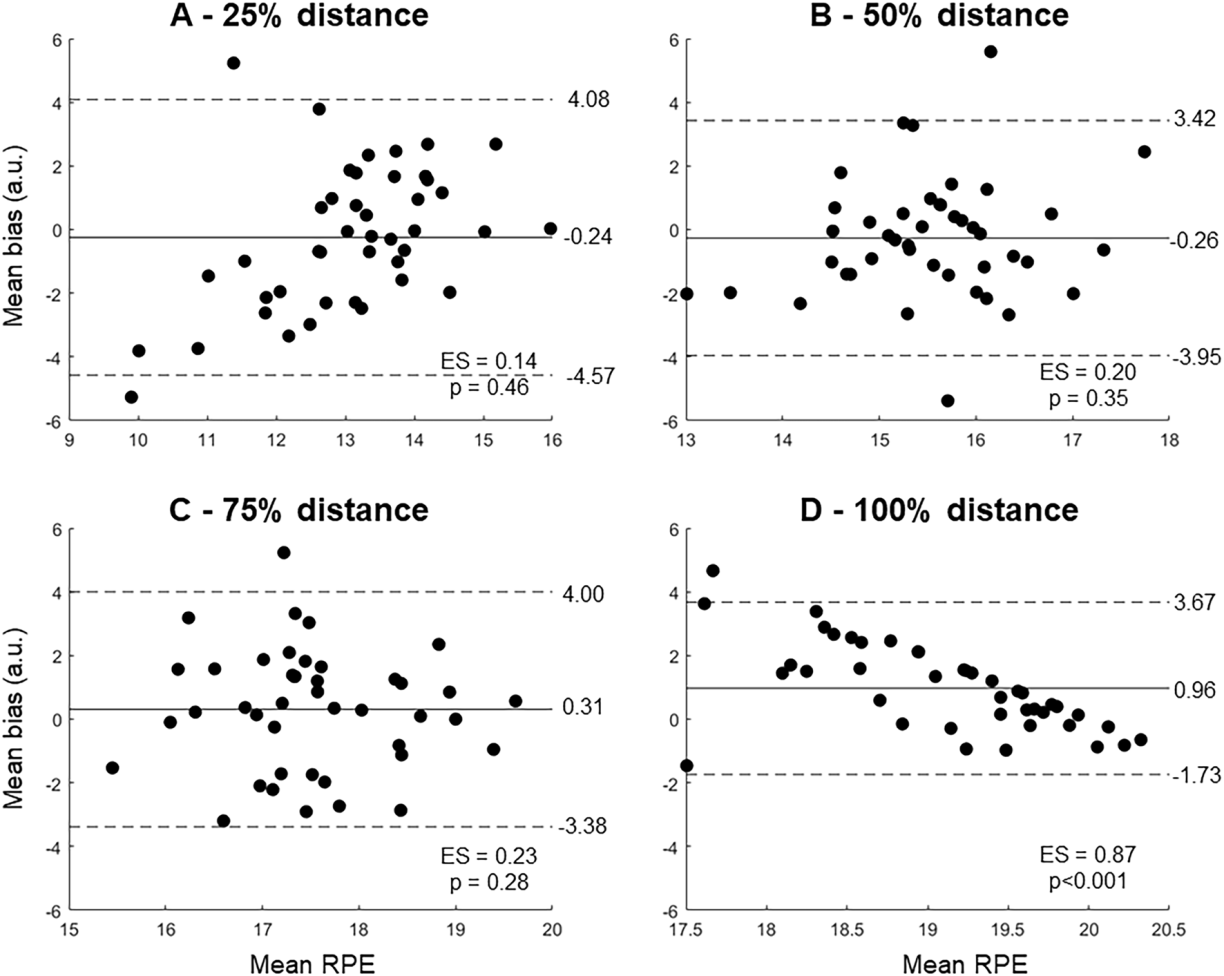
Bland–Altman plots for the predictions of ratings of perceived exertion (RPE) at 25% (**A**), 50% (**B**), 75% (**C**) and 100% of the duration of a 5-km simulated running race. The horizontal solid lines represent the mean bias, whereas the dashed lines represent the upper and lower limits of agreement (95% confidence intervals). The effect size (ES) and pairwise statistical difference (*p*) between real and predicted RPE are shown for each subplot

When the real and predicted RPE data were compared across 25%, 50%, 75%, and 100% of the running distance, it was found that the mean bias was nearly zero for 25% (Fig. 3A), 50% (Fig. 3B) and 75% of the running distance (Fig. 3C). These three percentages of running distances presented low effect sizes (~ 0.2) and no significant differences in the t Student pairwise comparisons (*p* < 0.05). However, the mean bias was increased to 0.96 for the 100% running distance (Fig. 3D), showing a large effect size (0.87) and a significant statistical difference between real and predicted RPE revealed by the *t* Student test. The last reported RPE was 19.7 ± 0.5, and ~ 80% of runners (34 out of 43) reported 20 on the RPE scale. Regarding the predicted RPE, the average RPE at the end of the exercise was 18.7 ± 1.3 and the RPE reached 20 for 34% of runners (15 out of 43).

### Subject-dependent models

**Fig. 4:**
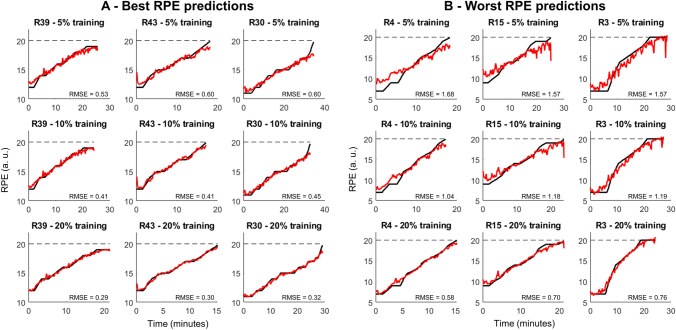
Real (black lines) and predicted rating of perceived exertion (RPE, red lines) during a 5-km simulated race from the best (**A**, left panel) and worse prediction performance (**B**, right panel) based on the root-mean-square error (RMSE). The columns represent the same runner (indicated as ‘R’ in the subplot titles), whereas the rows represent the predictions using 5% (top row), 10% (middle row), and 20% of the data for model training (bottom row)

The training of the subject-dependent model with only 5% of the total dataset required 1.2 ± 0.3 min of data, whereas this time increased to 2.4 ± 0.6 and 4.8 ± 1.2 min when using 10% and 20% of the total time, respectively. Since we used a balanced amount of data from each 25% of the total duration, it is required approximately 18 s of data from each of these 25% sectors when using only 5% of the total data. Figure 4 illustrates examples of RPE predictions using the three different subject-dependent models. The quality of the prediction when comparing the best (Fig. 4A) and worse predictions (Fig. 4B) is more similar when compared to the subject-independent models in Fig. 2. The subject-dependent models using only 5% of the data for training presented an average RMSE of 1.00 ± 0.31 (Table 1), whereas the average RMSE from the subject-independent model was 1.80 ± 0.81 (Table 1). Moreover, increasing the size of the training dataset from 5 to 10% or 20% of the total time increases the model performance for both Pearson correlations and RMSE parameters (Table 1). The coefficient of variation of the RMSE across the 10 different models applied with each amount of data was 3.2 ± 1.29%, 2.43 ± 0.94%, and 1.85 ± 0.73% when the model was applied with 5%, 10%, or 20% of the total data, respectively.

**Fig. 5:**
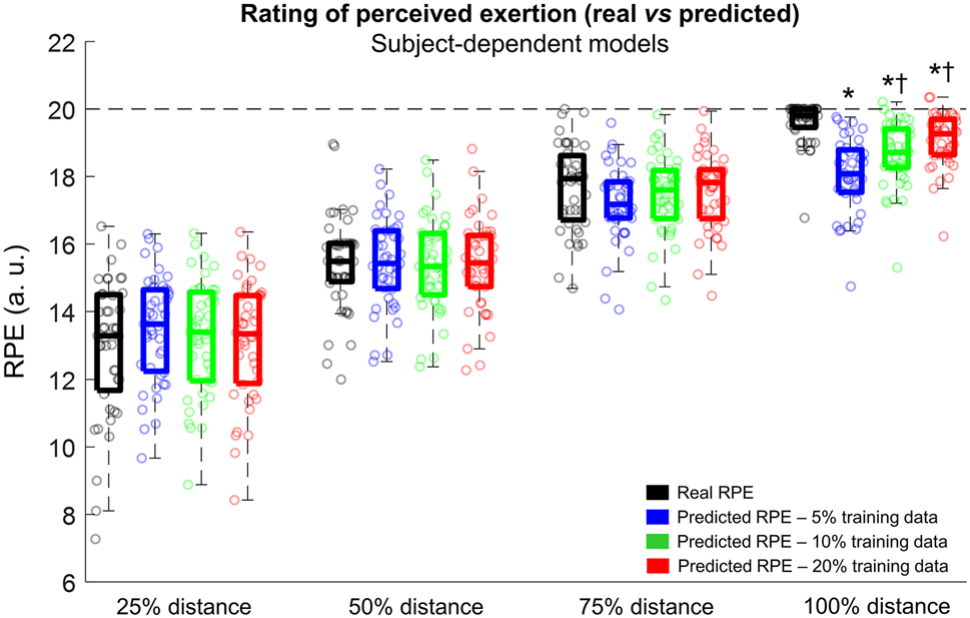
Real (black color) and predicted rating of perceived exertion (RPE) using 5% (blue color), 10% (green color), and 20% of training data (red color) at different stages of a 5-km simulated race. Boxplots represent 25th and 75th percentiles, and data range (dash vertical lines). Open circles represent individual runners from the sample. * denotes a significant difference in relation to the real RPE (*p* < 0.001). † denotes a significant difference in relation to the predicted RPE using 5% training data (*p* < 0.01)

Similarly to the subject-independent models, the mean bias was nearly zero and the effect size of comparisons was low when comparing the real and predicted RPE from 25%, 50% and 75% of the running distance, regardless of the amount of training data used (see Supplementary Fig. 2). However, the mean bias was greater than 0.5 and the effect sizes were larger than predictions at 100% of the running distance (see Supplementary Table 1). The statistical analysis using a two-way repeated measures ANOVA corroborated such results, demonstrating no differences between real and the three prediction methods at 25%, 50%, and 75% of the total running distance (Fig. 5, *p* > 0.05). However, there was a significant effect of the extraction method at 100% of the total running distance (*F*(3,168) = 24.2, *p* < 0.0001, effect size = 0.30), where the real RPE was significantly higher when compared to the predictions using 5%, 10% and 20% training data (*p* < 0.001). In addition, the predicted RPE using 5% training data was significantly lower when compared to the predictions with 10% (*p* < 0.01) and 20% training data (*p* < 0.001).

## Discussion

The main finding of the present study was that running biomechanical variables extracted from a commercially available smartwatch paired with a chest strap allows the prediction of perceived exertion within an average of 1.8 RPE points error (median = 1.5 RPE points) when using subject-independent machine learning models. Moreover, the prediction error reached an average of ~ 1 RPE point when using only 5% of the data for training the model to predict the RPE of a runner (~ 80 s running at different fatigue levels), while the prediction error was on average below 0.5 RPE point when using 20% of the data for training. However, there was a predominant underestimation of the RPE at the end of the exercise, regardless of the type of machine learning model applied. These results suggest that running biomechanical data that are highly accessible through commercial smartwatches can be used to provide external feedback regarding subjective fatigue levels to running practitioners. Therefore, future products targeting running practitioners may implement fatigue tracking throughout running workouts to assist runners to dose their efforts, both to improve performance and potentially reduce injury risks.

A previous study (De Beéck et al. 2018) explored the prediction of RPE during outdoor running at various running speeds using machine learning models based on inertial measurement units (IMUs) data from the wrist, arm, and tibia. The study evaluated both subject-independent and dependent models using gradient-boosted regression trees, artificial neural networks, and linear regressions. The authors extracted 200 features per IMU and predicted RPE using single IMUs or combinations of two or three IMUs, reaching predominantly high accuracy in predicting RPE (mean absolute error < 2.5, no variability reported). In comparison, the mean absolute error from our subject-independent prediction was 10.5 ± 6, and our greater error may be related to the use of only six features extracted from a single accelerometer. Nonetheless, the purpose of our study was to demonstrate the feasibility of using data from a commercially available wearable sensor to predict RPEs, while requiring no preprocessing other than feature scaling and a 5-s window average. From a practical point of view, the present study provides a highly accessible method to implement feedback in running workouts.

In another study (Gholami et al. 2020), machine learning was used to predict RPEs during treadmill running using mechanical strain data from a textile wearable trouser. The study used a 2-step random forest regressor algorithm with 100 ensemble trees and 24 features. The results showed high correlation (*r*^2^ = 0.96) and low absolute error (RMSE = 0.06 RPE points) across five female runners. As a comparison, our RMSE was 0.39 RPE points when using 20% training data across 43 runners. It is noteworthy that the study from Gholami and co-workers (Gholami et al. 2020) evaluated a homogeneous sample of only five female runners with similar performance during a 10-km race that could fit the prototype garment. Conversely, our sample consisted of a more heterogeneous sample in terms of gender, training status, and anthropometric characteristics. Nonetheless, their findings are encouraging for the use of objective measurement of fatigue through wearable technology.

Regarding longer distance running, Marotta and co-workers used a machine learning three-level classifier to predict fatigue levels from eight healthy runners during a 4000-m run (Marotta et al. 2021). A total of 157 features were extracted from 8 IMUs located on the sternum, pelvis, right and left thigh, tibia, and leg. The authors explored different IMU combinations from single sensors to all sensors, and the single-sensor prediction may be comparable to our setup. The single-sensor prediction used a sensor on the tibia and extracted 12 features to classify the effort as minimal, mild, or maximum. The classification accuracies were 76.5%, 65.4%, and 86.3% for minimal, mild, and maximum efforts, respectively. Our results cannot be directly compared to those from Marotta and co-workers since we investigated regression models. However, both studies demonstrate that data from wearable sensors such as IMUs allow for moderately accurate fatigue predictions and may be a step toward using IMU data to determine training loads.

During steady-state exercises, it is expected that RPE steadily increases, whereas neuromuscular fatigue compromises motor performance concomitantly (Skatrud-Mickelson et al. 2011). In our study, the exercise intensity (e.g., running speed) was constant throughout the experiment, but fatigue steadily increased due to the long exposure to the same exercise intensity. The exercise was terminated when the runner could not maintain the proposed pace, reaching exercise failure that may correspond to the maximum exertion for the running intensity. Interestingly, the real RPE progression pattern varied widely across runners, with some runners presenting steady periods with the same RPE at the beginning and/or at the end of the exercise. If one considers that fatigue is an incremental process at a constant exercise intensity, the reporting of invariant RPE throughout several minutes seems controversial despite being a subjective metric. The use of machine learning to predict RPE revealed that runners presenting extended periods of constant RPE presented worse predictions (see Fig. 2). The inferior prediction quality may be related to a constant impairment of motor performance captured by the biomechanical data, whereas the runner did not “feel” the performance decay and reported identical numbers within the same period. Therefore, the biomechanical dataset can detect fatigue-related changes in motor behavior while the subjective RPE may not be appropriate to represent the fatigue progression.

The feeling/sensation experienced during strenuous exercise may induce a greater perception of fatigue. It is known that metabolite accumulation during fatiguing exercises induces generalized acidosis that might cause waves of nausea and other symptoms (Samborski et al. 2013), especially in recreational runners. Therefore, the perception of general discomfort can be extreme, and the overall perceived exertion is at the maximum. In addition to extreme discomfort, it is plausible that the motor system can still deliver running-like motion patterns despite substantial peripheral fatigue, preventing predictions based on running biomechanical data to reach RPE closer to 20 for the majority of runners. Therefore, it is plausible that RPE predictions based on biomechanical features will underestimate extreme fatigue since the running biomechanical features will capture the objective motor performance rather than the subjective exertion experienced.

As expected, subject-dependent models reached higher accuracy in predicting RPE when compared to the subject-independent model, and the prediction quality was directly related to the amount of data allocated to train the models. The average root-mean-square error using only 5% of data for training was 1.00 ± 0.31 (relative error ~ 6%). The superior performance of subject-dependent models is related to the RPE predictions being based on a sub-sample of running biomechanical data from the same runner, drastically reducing the dataset variability. Nonetheless, the subject-independent models reached relative errors below 12% on average. The results of our study may indicate that the implementation of automatic predictions of perceived exertion may be more reliable when using subject-dependent models. Moreover, it is possible to reach prediction accuracy within 1 RPE point with the use of a few minutes of running data. In practice, a calibration session may be required to provide fresh and fatigued running mechanics data into a prediction algorithm, while the runner only needs to keep different fatigue stages for ~ 20 s. Future studies evaluating different running intensities, as well as exercises with varying running speeds are necessary to further develop models that can adapt to runner’s training routines and physical capabilities.

Previous studies have been exploring RPE predictions during running using machine learning techniques, although with major differences when compared to our study. In particular, the number of features used in previous studies is substantially larger, ranging from 24 (Gholami et al. 2020) to > 100 features (De Beéck et al. 2018; Marotta et al. 2021). A greater number of features may be advantageous to improve model accuracy, but our study focused on the use of a handful of features highly accessible through a fitness smartwatch. Despite a lower accuracy was seen compared to the previously mentioned studies, our results are a remarkable step toward true implementation of fatigue prediction using wearable sensors. Although accessing running biomechanical features is currently dependent on the use of chest straps the field may highly benefit from further research using data from the accelerometer located in the smartwatch itself. The accelerometer fixation on the wrist or waist can cause discrepancies in step count in both laboratory and free-living conditions (Tudor-Locke et al. 2015). However, its future studies should explore the creation of similar prediction models for fatigue using the wrist accelerometer sensor and machine learning.

## Conclusion

In summary, the present study demonstrated the feasibility of predicting a runner’s RPE from only six running biomechanical data features extracted from a commercially available smartwatch/chest strap. RPE predictions based on other runner’s data (subject-independent models) reached accuracies below 2 RPE points, whereas predictions based on the same runner’s data (subject-dependent models) reached accuracies at ~ 1 RPE point using less than 90 s of running data to predict RPE throughout more than 20 min. In-built algorithms to provide fatigue status during running workouts seem possible using both types of prediction, but there are underestimations of the RPE values closer to or at the maximum exertion (RPE = 20).

### Supplementary Information

Below is the link to the electronic supplementary material.

Supplementary Fig. 1 (DOCX 45 KB)

Supplementary Fig. 2 (DOCX 105 KB)

Supplementary Table 1 (DOCX 14 KB)

## Data Availability

The data supporting the findings described in this study are available on request from the corresponding author.
