# Supplementary material for: Prediction of instantaneous perceived effort during outdoor running using accelerometry and machine learning
Source: Eur J Appl Physiol. 2023 Sep 29;124(3):963–73. doi: 10.1007/s00421-023-05322-0 (PMC10879226; doi:10.1007/s00421-023-05322-0)
Supplement: Supplementary file 1 — Supplementary Fig. 1 (DOCX 45 KB) [file 421_2023_5322_MOESM1_ESM.docx]

**
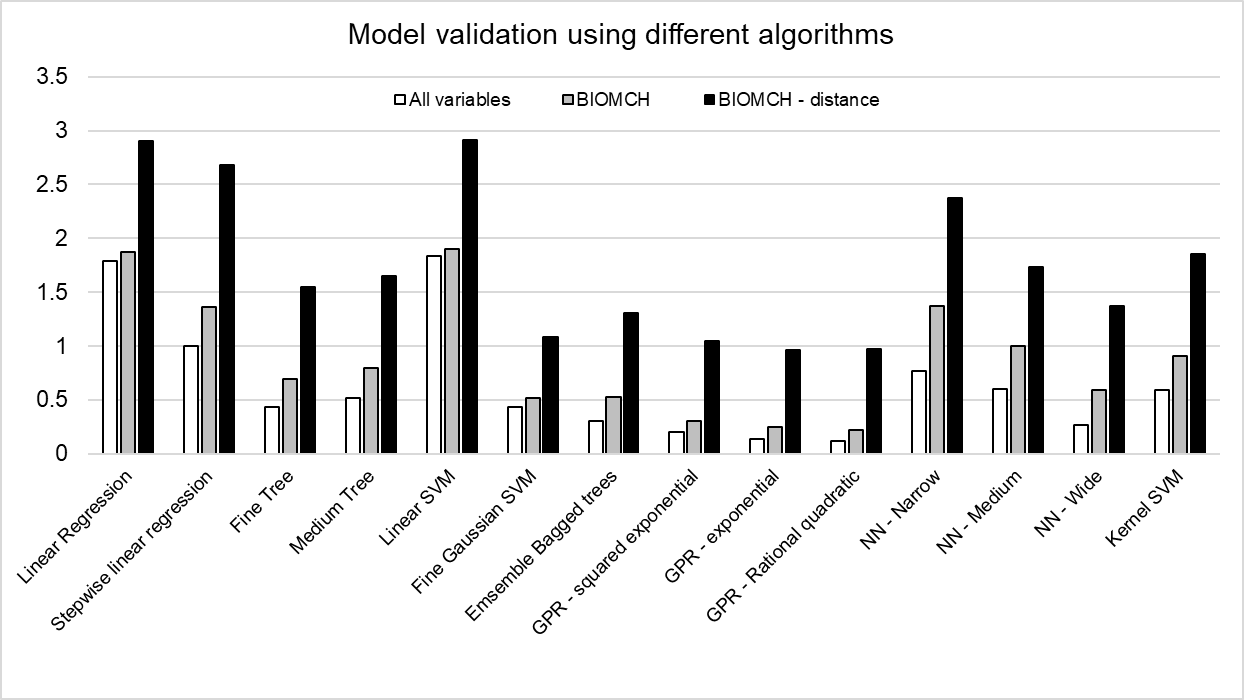
**

**Supplementary Figure 1.** Prediction error (in RPE points) when applying different machine learning algorithms to predict RPE using age, body mass and body height combined with running biomechanical data (All variables, *white bars*), only biomechanical data (BIOMCH, *gray bars*) and only biomechanical data except running distance (BIOMCH – distance, *black bars*).SVM = support vector machine; GPR: gaussian process regression; NN = 2-layer neural network.
