# Supplementary material for: Prediction of instantaneous perceived effort during outdoor running using accelerometry and machine learning
Source: Eur J Appl Physiol. 2023 Sep 29;124(3):963–73. doi: 10.1007/s00421-023-05322-0 (PMC10879226; doi:10.1007/s00421-023-05322-0)
Supplement: Supplementary file 2 — Supplementary Fig. 2 (DOCX 105 KB) [file 421_2023_5322_MOESM2_ESM.docx]

*
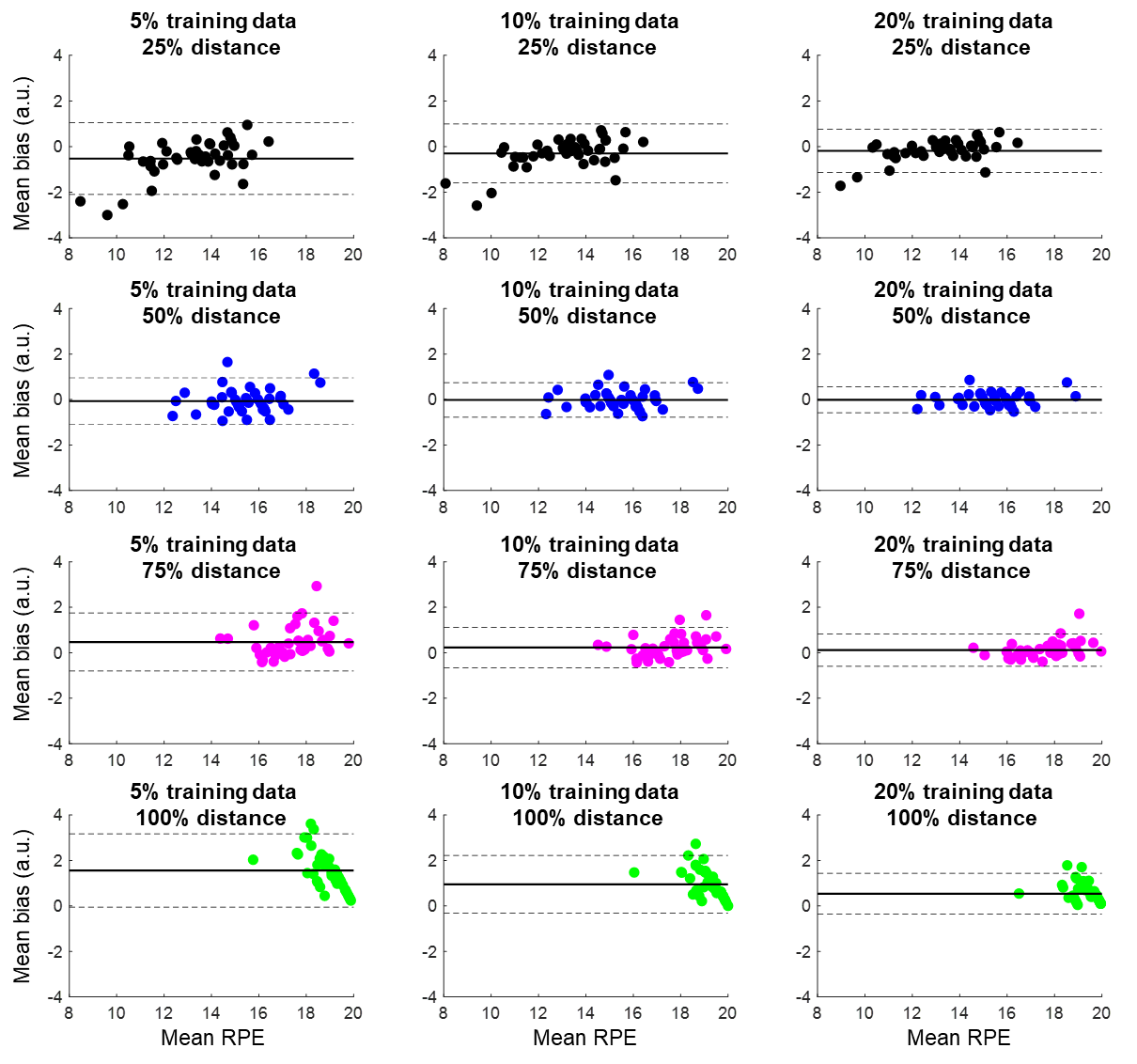
*

**Supplementary Figure 2**. Bland-Altman plots for the predictions of ratings of perceived exertion (RPE) at different running distances (each row represents a difference, with individual data points illustrated in different colors), and using different amounts of data for training the machine learning algorithm (each column represents either 5%, 10% or 20% of the data used for training). The horizontal solid lines represent the mean bias, whereas the dashed lines represent the upper and lower limits of agreement (95% confidence intervals). The effect size (ES) and pairwise statistical difference (p) between real and predicted RPE are shown for each subplot.
