# Supplementary material for: Prediction of instantaneous perceived effort during outdoor running using accelerometry and machine learning
Source: Eur J Appl Physiol. 2023 Sep 29;124(3):963–73. doi: 10.1007/s00421-023-05322-0 (PMC10879226; doi:10.1007/s00421-023-05322-0)
Supplement: Supplementary file 3 — Supplementary Table 1 (DOCX 14 KB) [file 421_2023_5322_MOESM3_ESM.docx]

**Supplementary Table 1.** The effect size, mean bias and upper/lower limits of agreement (LOA, 95% confidence intervals) from the comparison between real and predicted RPE at different running distances (dist) and using different amounts of data for the training datasets (TR Data).

| **Conditions** | **Effect size** | **Mean bias** | **Lower bound**  **LOA** | **Upper bound**  **LOA** |
| --- | --- | --- | --- | --- |
| **25% dist / 5% TR Data** | -0.29 | -0.53 | -2.10 | 1.04 |
| **25% dist / 10% TR Data** | -0.16 | -0.30 | -1.59 | 0.99 |
| **25% dist / 20% TR Data** | -0.10 | -0.19 | -1.14 | 0.77 |
| **50% dist / 5% TR Data** | -0.05 | -0.07 | -1.09 | 0.95 |
| **50% dist / 10% TR Data** | -0.01 | -0.02 | -0.78 | 0.74 |
| **50% dist / 20% TR Data** | -0.01 | -0.01 | -0.58 | 0.56 |
| **75% dist / 5% TR Data** | 0.39 | 0.47 | -0.80 | 1.74 |
| **75% dist / 10% TR Data** | 0.19 | 0.23 | -0.66 | 1.12 |
| **75% dist / 20% TR Data** | 0.10 | 0.12 | -0.59 | 0.83 |
| **100% dist / 5% TR Data** | 1.33 | 1.56 | -0.05 | 3.17 |
| **100% dist / 10% TR Data** | 1.03 | 0.95 | -0.31 | 2.22 |
| **100% dist / 20% TR Data** | 0.71 | 0.53 | -0.37 | 1.43 |
